# Gut microbiota–M cell co-culture in inflammatory bowel disease and its therapeutic potential in organoid platforms

**DOI:** 10.3389/fphar.2026.1778142

**Published:** 2026-04-20

**Authors:** Siyuan Zuo, Hiroki Kurumi, Ryohei Ogihara, Tsutomu Kanda, Hajime Isomoto

**Affiliations:** Division of Gastroenterology and Nephrology, Faculty of Medicine, Tottori University, Yonago, Japan

**Keywords:** gut microbiota, IBD, medicinal potential, microfold cell, organoid

## Abstract

Dysbiosis of the gut microbiota is a key driver in the onset and persistence of inflammatory bowel disease (IBD). However, the mechanisms by which microbes influence mucosal immunity via specific epithelial routes remain incompletely elucidated. Microfold (M) cells within follicle-associated epithelium serve as a critical “gateway” for luminal antigens and microbes to access the mucosal immune system. While essential for surveillance of commensal microbes, M cells could also be exploited by adherent-invasive strains and adverse environmental factors to amplify inflammation. Recent studies suggest that both in Crohn’s disease and ulcerative colitis, M cell abundance and function are aberrantly regulated, linking microbial imbalance with heterogeneous mucosal inflammatory phenotypes. Traditional animal models and two-dimensional culture systems retain limited capacity to selectively manipulate M cells without perturbing systemic immunity, thereby constraining systematic studies of microbiota–M cell co-cultures. Advances in intestinal organoid technology now enable controlled induction of functionally mature M cells within three-dimensional epithelial structures, and have started to shed light on the roles of RANKL signaling, negative regulators, and microbe-associated factors in M cell differentiation and homeostasis. In this review, we focused on key evidence supporting microbiota–M cell interactions in IBD, discussed how M cell-enriched intestinal organoid models could be leveraged to dissect the impact of pathogenic microbes, candidate probiotics, dietary components, and existing therapies on these interactions as well as to evaluate the related potential and limitations for microbiome interventions and drug screening. Integrating gut microbial plasticity with M cell epithelial entry and organoid platforms promises to provide new experimental foundations and theoretical support for individualized microbiome-based therapies and targeted mucosal treatments in IBD.

## 1 Introduction

The gut microbiota is crucial for maintaining mucosal homeostasis, and disruptions in its composition and function have been linked to the development and persistence of inflammatory bowel disease (IBD) (Pascal et al., 2017; Lloyd-Price et al., 2019). With accelerating global industrialization, the prevalence of IBD continues to rise and has become an important global public health burden (Hracs et al., 2025; Kaplan, 2025). IBD encompasses Crohn’s disease (CD) and ulcerative colitis (UC), chronic relapsing inflammatory conditions of the intestine influenced by complex interactions among genetic susceptibility, environmental factors, microbial dysbiosis, and immune dysregulation. However, the precise underlying mechanisms driving disease remain unclear (Yang and Jostins-Dean, 2022; Xue et al., 2024).

Microfold (M) cells are specialized epithelial cells located in the follicle-associated epithelium covering Peyer’s patches and intestinal lymphoid follicles, functioning in luminal antigen sampling and immune induction. Classical studies, largely based on murine infection models and preclinical systems, have demonstrated that the gut microbiota could translocate across the epithelium via M cells, triggering immune responses and potentially initiating intestinal inflammation (Kalischuk et al., 2010; Chassaing et al., 2011; Rios et al., 2015). In certain patients with, early lesions are frequently located in ileal Peyer’s patch epithelium enriched for M cells, where microbial translocation is considered a potential focal trigger (Roberts et al., 2010; Vazeille et al., 2016). In UC, although inflammation predominantly involves the mucosal layer, expansion of M cells in colonic lamina propria lymphoid follicles may increase antigen load and sustain inflammation (Bennett et al., 2016; PEREZ-JELDRES et al., 2020).

**FIGURE 1:**
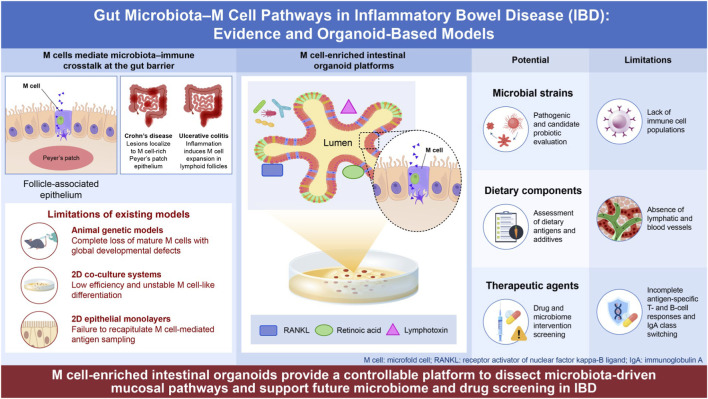
Gut Microbiota–M Cell Pathways in Inflammatory Bowel Disease (IBD): Evidence and Organoid-Based Models. Schematic overview of M cell–mediated microbiota–immune interactions in IBD and the application of M cell–enriched intestinal organoid platforms. The schematic integrates interactions supported by experimental evidence together with proposed or putative pathways based on emerging studies.

However, M cells are rare, their selective manipulation is difficult, and traditional animal models and two-dimensional culture systems retain limited utility for mechanistic study of microbiota-mediated immune effects via M cells (Kanaya et al., 2018). The advent of intestinal organoid (enteroid) technology has enabled the induction of functional M cells within three-dimensional epithelial structures (Rouch et al., 2016; Wood et al., 2016). More recently, human organoid systems have confirmed that mature GP2^+^ M cells could be induced by retinoic acid and lymphotoxin signals, providing a powerful *in vitro* platform for studying differentiation mechanisms and microbe interactions (Ding et al., 2020; Luna Velez et al., 2023). To provide a visual summary of the proposed interactions between gut microbiota and M cells in the pathogenesis of inflammatory bowel disease (IBD), including both experimentally supported mechanisms and emerging hypothetical pathways, and to illustrate the corresponding organoid-based experimental approaches, we present a schematic representation in Figure 1.

Although murine studies have provided important mechanistic insights into M-cell differentiation, antigen sampling, and microbe–epithelium interactions, direct extrapolation of these findings to human intestinal biology should be made with caution. Human M cells differ from murine counterparts in tissue context, inducible programs, and potentially in immune-interaction capacity, as suggested by recent human organoid-based studies. In this review, we therefore prioritize human evidence where available, while discussing murine findings primarily as mechanistic support or model-derived context.

In this mini-review, we summarized the currently available key evidence for microbiota–M cell interactions in IBD pathogenesis, and highlights applications and limitations of M cell-enriched intestinal organoid models for investigating microbe–epithelium crosstalk, thereby validating candidate probiotics or dietary factors, and supporting drug and microbiome intervention screening.

### 1.1 Gut microbiota dysbiosis in IBD: From multi-omics evidence to interventions

Longitudinal multi-omics cohort studies consistently show that the gut microbiota in patients with IBD undergoes marked changes in both community structure and functional activity. In the multi-omics IBD resource generated by the Integrative Human Microbiome Project (iHMP), patients with Crohn’s disease and ulcerative colitis, compared with healthy controls, exhibit a significant reduction in α diversity, accompanied by an expansion of facultative anaerobes (mainly Proteobacteria such as *Escherichia coli*) and a pronounced depletion of strictly anaerobic, short-chain fatty acid (SCFA)–producing taxa (including Faecalibacterium prausnitzii, Roseburia spp., and Eubacterium rectale). These compositional shifts are closely associated with coordinated changes in microbial transcriptional activity, remodeling of the metabolite pool, and host immune status, including reduced secondary bile acids, lower butyrate levels, and increased mucosal inflammation markers (such as C-reactive protein and fecal calprotectin). Together, these findings suggest a tight coupling between microbial dysbiosis and inflammatory activity (Lloyd-Price et al., 2019).

Traditional metagenomic analyses alone have limited ability to capture functional changes at the activity level, but metatranscriptomic data provide complementary evidence. Schirmer et al. performed combined metagenomic and metatranscriptomic analyses of fecal samples from patients with IBD and found that the gene expression patterns of certain microbial species during inflammation differed significantly from their gene abundance, suggesting that alterations in functional activity may be more directly associated with inflammatory states than mere species presence (Schirmer et al., 2018).

Further longitudinal integrative studies suggest that the gut microbiota not only reflects disease status but also has potential predictive value. A recent cohort study of pediatric ulcerative colitis published in Nature Communications integrated mucosal microbiome, host epigenome, and transcriptome data. The authors found that enrichment of the oral-derived bacterium Veillonella parvula was significantly associated with subsequent disease relapse, whereas depletion of butyrate-producing bacteria was closely coupled with activation of the host IFN-γ signaling pathway. A machine-learning model constructed from the combined multi-omics dataset showed markedly better performance in predicting relapse than models based on any single omics layer. These findings indicate that microbial dysbiosis may directly participate in the regulation of intestinal inflammation through specific immune pathways, rather than serving merely as a passive biomarker (Kulecka et al., 2025).

At the same time, the modulatory role of external environmental factors on this host–microbe interaction axis is increasingly supported. Recent longitudinal cohort data show a significant inverse association between a dietary quality index and intestinal inflammatory activity, with part of this effect mediated through microbiome-related metabolic pathways. For example, high-fiber diets can enhance pathways for butyrate and propionate production and suppress the conversion of primary bile acids into pro-inflammatory secondary bile acids. In contrast, high-fat diets are associated with expansion of sulfate-reducing bacteria such as Bilophila wadsworthia and increased hydrogen sulfide production, thereby exacerbating epithelial barrier damage and mucosal inflammatory responses. These studies highlight microbial metabolic pathways as key intermediates linking environmental exposures to disrupted intestinal immune homeostasis (Mayorga et al., 2026).

In interventional studies, specific microbiota features reportedly predicted therapeutic responses. Intensive multi-donor fecal microbiota transplantation (FMT) in randomized controlled trials has induced steroid-free clinical and endoscopic remission in approximately one-third of patients with active UC, accompanied by restoration of specific commensal communities (Paramsothy et al., 2017). Compared with total enteral nutrition, the Crohn’s disease exclusion diet combined with partial enteral nutrition had a comparable remission rate but better tolerance in children with CD, and the fecal microbiota composition was closer to that of healthy samples (Levine et al., 2019) In addition, Lee et al. evaluated the association between the gut microbiome and response to biologics (such as anti-TNFα agents) in patients with IBD and found that particular microbial patterns were significantly associated with treatment outcomes, suggesting that the microbiota may serve not only as a disease marker but also as a determinant of therapeutic efficacy (Lee et al., 2021).

Importantly, these multi-omics and longitudinal studies do more than refine the descriptive landscape of dysbiosis in IBD; they also expose several unresolved mechanistic gaps that may be particularly amenable to testing in M cell–enriched organoid systems. First, it remains unclear whether relapse-associated microbial shifts and metabolic perturbations act as upstream drivers of inflammation or arise secondarily from an already inflamed mucosal environment. Second, these datasets do not resolve through which epithelial routes luminal dysbiosis is functionally translated into mucosal immune activation. In particular, whether specific microbes or microbiota-derived metabolites promote inflammation in part by altering M cell differentiation, antigen uptake, or transcytosis remains largely unknown. Third, the causal relevance of relapse-associated signatures, including depletion of butyrate-producing taxa and enrichment of Veillonella-associated profiles, cannot be established from association studies alone. These unresolved issues highlight the need for experimentally tractable human epithelial platforms in which microbial strains, metabolites, and inflammatory cues can be introduced in a controlled manner to directly test M cell–dependent mechanisms.

### 1.2 M cells: A “double-edged sword” for microbial and antigen entry into mucosal immunity

M cells are follicle-associated epithelial cells located on the surface of small intestinal Peyer’s patches and colonic isolated lymphoid follicles, characterized by sparse microvilli, a cup-shaped apical surface, and a basolateral “pocket-like” invagination accommodating dendritic cells and B cells (Ohno, 2016; Kanaya et al., 2018). M cells express GP2, recognizing type I fimbriae (FimH) of Gram-negative bacteria, allowing for both commensal and pathogenic bacteria to be efficiently internalized and transcytosed across the epithelium (Hase et al., 2009; Ohno and Hase, 2010). Using an *ex vivo* human Peyer’s patch M cell co-culture model, Roberts et al., further demonstrated that AIEC translocation across M cells could be significantly inhibited by soluble psyllium fiber, whereas the food emulsifier polysorbate 80 enhances this process (Roberts et al., 2010). Taken together, M cells form a critical node linking microbial signals and dietary influences. Under homeostatic conditions, they facilitate immune education, whereas in the case of microbial or environmental imbalance, they might become a convergence point through which pathogens and dietary factors jointly drive inflammation.

**FIGURE 2:**
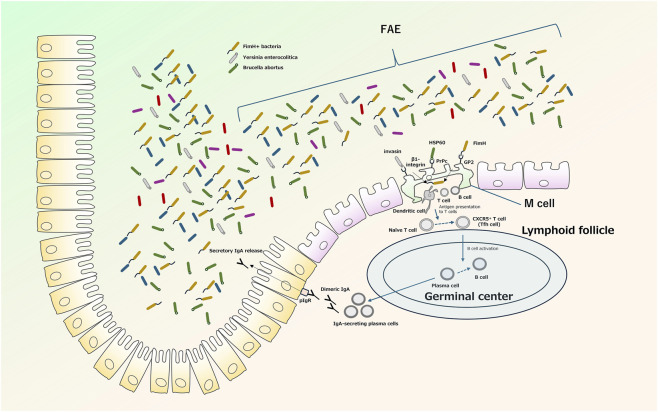
Schematic overview of M cell-mediated antigen uptake and mucosal immune induction in the follicle-associated epithelium M cells within the follicle-associated epithelium (FAE) take up luminal microorganisms and macromolecular antigens and transport them across the epithelial barrier to the underlying lymphoid tissue. Representative receptor–ligand interactions shown here include FimH–GP2, invasin–β1-integrin, and HSP60–PrP(C). Antigen delivery to subepithelial immune cells supports antigen presentation, T-cell and B-cell activation, germinal center reactions, plasma-cell differentiation, and secretory IgA production. This schematic summarizes major concepts relevant to M cell-dependent mucosal immune surveillance.

Emerging studies indicate that intestinal M cells may play context-dependent roles beyond simple transcytosis of luminal microbes. In murine models, antigen sampling by M cells is a critical initiator of secretory IgA responses and mucosal tolerance to commensal bacteria, with genetic ablation of RANKL-dependent M cell differentiation markedly delaying Peyer’s patch germinal center maturation and IgA plasma cell emergence in mice, leading to diminished fecal SIgA and impaired homeostasis (Michaud et al., 2022). However, under chronic inflammatory contexts typical of IBD, excessive or aberrant M cell activity has been associated with increased microbial translocation and pro-inflammatory signals in murine models, suggesting a potential role in amplifying mucosal inflammation. Moreover, recent human organoid studies reveal that human M cells not only transport antigens but also express MHC class II molecules and dendritic cell-related transcriptional programs, enabling direct antigen presentation independent of IFNγ stimulation—a feature not shared with murine enterocytes and underscoring inter-species differences in M cell biology (Wang et al., 2025). While M-cell expansion in animal models is frequently linked to heightened inflammatory responses, human data indicate potential functional heterogeneity and context-dependent regulation of M-cell activity. While receptor-mediated uptake pathways have been well characterized in classical pathogen studies, much of this framework derives from murine or reductionist models and should be interpreted cautiously in the context of human IBD. As summarized in Figure 2, intestinal M cells within the follicle-associated epithelium form a specialized antigen-entry route that links luminal microbial recognition to downstream mucosal immune activation, including lymphoid follicle responses and secretory IgA production.

The longstanding view of M cells as predominantly pathogenic in IBD likely reflects the limitations of inflammation-biased animal models, rather than their full spectrum of functions in the human intestinal epithelium.

### 1.3 M cell abnormalities in IBD: Crohn’s disease and ulcerative colitis

In Crohn’s disease, clinicopathological observations revealed that the ileal Peyer’s patch overlying epithelium is a common site for early ulceration and inflammation. Human- and disease-relevant experimental studies have demonstrated that AIEC, via its long polar fimbriae (LPF), binds to GP2 expressed on the surface of M cells and promotes intestinal microbe translocation from the lumen into the lymphoid tissue beneath Peyer’s patches. Anti-GP2 antibody application significantly reduces these microbe–tissue interactions and translocation events, providing mechanistic evidence for microbial entry via this epithelial route (Chassaing et al., 2011). Beyond its role as a transcytosis receptor, GP2 expression is markedly upregulated in the FAE of activeCD lesions. Together with the spatially reorganized distribution of other epithelial subsets (for example, Clu^+^ cells), this pattern forms a local cell–cell interaction network that can be resolved by spatial transcriptomics to distinguish inflamed regions from adjacent, relatively normal tissue (Cadinu et al., 2024). These high-resolution data suggest that, in CD, M cells not only increase in number but also undergo inflammation-driven remodeling of their gene-expression programs and intercellular communication signals (Capeling et al., 2026). Although existing single-cell RNA-seq and spatial omics studies have generated detailed maps of epithelial and immune cell states in IBD tissues, the classification of intrinsic M-cell subpopulations is still at an early stage. Consistently, integrated single-cell and spatial omics analyses reveal a broadly remodeled epithelial–immune interaction landscape in CD, These changes are particularly evident in Peyer’s patches and lymphoid follicle regions, together with inflammation-driven reshaping of epithelial lineage programs, which may involve shifts in M-cell states (Huck et al., 2025). Similar alterations have also been observed in the ileal FAE, although the fine-grained resolution of M cell subpopulations remains limited (Kong et al., 2023). Moreover, large-scale single-cell RNA-sequencing analyses show that IBD tissues commonly display broad interferon responses and altered cell–cell communication across both epithelial and immune compartments. These network-level disruptions may act together within the inflammatory cycle to promote activation of M-cell–related pathways (Thomas et al., 2024).

In ulcerative colitis, direct human evidence for stable expansion of *bona fide* M cells remains limited, although human single-cell studies support the expansion of microfold-like epithelial states and M-cell-associated transcriptional programs (Smillie et al., 2019; Jagadeesh et al., 2022). Complementary evidence from mouse colitis models further suggests that large numbers of GP2^+^ “Peyer’s patch-like” M cells appear in the FAE overlying colonic isolated lymphoid follicles, concomitant with upregulation of local TNFα and RANKL (Bennett et al., 2016). In murine colitis models, Parnell et al. further demonstrated that inflammation-induced colonic M cells depend on TNFR2 rather than LTβR; in TNFR2-deficient mice, colonic M cells are barely induced even when colitis is present (Parnell et al., 2017). Together, these murine data raise the possibility that sustained inflammatory signals may drive inducible M-cell programs in chronic colonic inflammation; however, whether the same process occurs in human UC tissues remains to be established. Notably, extrapolating from preclinical studies, anti-TNFα biologics may secondarily restrain inflammation-associated colonic M-cell induction; however, direct evidence that this reduces M-cell-mediated antigen uptake in human UC remains lacking (Bennett et al., 2016; Parnell et al., 2017). In addition, scRNA-seq datasets that specifically focus on M cells in UC are still limited, possibly because the colon has a low baseline density of M cells. However, integrated single-cell omics data show that UC epithelial cells display strong IFN-responsive and inflammatory core programs, including interferon-response signatures and increased expression of injury-associated genes. This suggests that epithelial states induced during chronic inflammatory cycles may also shape M-cell–like lineages and their capacity for antigen transcytosis (Thomas et al., 2024).

### 1.4 Model gap: Limitations of traditional animal models and 2D systems

Traditional gene knockout models exhibit substantial technical limitations in dissecting M cell function. For example, epithelial-specific deletion of TRAF6 leads to complete loss of mature M cells in the FAE and widespread silencing of FAE-associated gene expression, not only eliminating M cells themselves but also disrupting the entire immune sampling structure, thereby making the distinction of “M cell loss *per se*” effects from those arising from “global epithelial/immune developmental defects” difficult (Kanaya et al., 2018). In addition to TRAF6-deficient models, Spi-B knockout mice, which lack mature M cells due to disruption of the RANKL–Spi-B axis, have demonstrated the importance of M cells in antigen sampling and IgA induction. However, because these models completely lack M cells, they fail to recapitulate the inducible and dynamic nature of M cell differentiation observed in human IBD (Nakamura et al., 2020).

Although the commonly used Caco-2/Raji B co-culture system attempts to induce M cell-like properties in a 2D monolayer *in vitro*, such models retain clear limitations. Although co-culture could generate epithelial cells displaying certain M cell markers and functions, the differentiation efficiency remains low, phenotypes are unstable, and mature M cell states could not be maintained for long. More importantly, this monolayer epithelium type could not effectively support complex interactions among the intestinal microbiota and immune cells (MASUDA et al., 2011; Lapthorne et al., 2012).

Furthermore, traditional 2D epithelial models struggle to mimic chronic and selective antigen permeability. However, when exposed to complex microbial communities, Caco-2 monolayers typically lose epithelial integrity rapidly, resulting in loss of controlled barrier function. Nonetheless, these models cannot reproduce the three-dimensional structure and multi-cellular interaction environment necessary for recapitulating the authentic M cell-mediated antigen sampling process (Kollmann et al., 2023).

Classical pathogen studies in murine or reconstituted M-like systems have also provided important mechanistic clues regarding how luminal microbes exploit M-cell uptake pathways. For example, cellular prion protein (PrP(C)) is preferentially expressed on the apical membrane of Peyer’s patch M cells, and *Brucella abortus* has been shown to exploit PrP(C)-dependent uptake for entry into Peyer’s patches (Nakato et al., 2009; 2012). In the broader *Brucella* literature, heat shock protein 60 (Hsp60) has been identified as a PrP(C)-binding bacterial factor, supporting the concept that the Brucella–PrP(C) axis represents a receptor-mediated M-cell invasion pathway (Watarai et al., 2003). Likewise, β1-integrin has long been implicated in M-cell-mediated uptake of *Yersinia* enterocolitica, in reconstituted M-like epithelial models, apically expressed β1-integrin supports invasin-dependent internalization and translocation, although subsequent work suggests that additional invasin-independent bacterial determinants may also contribute to apical adhesion (Schulte et al., 2000; Hamzaoui et al., 2004). While these studies helped define receptor-level principles of M-cell-mediated uptake, their direct relevance to human IBD remains uncertain and they are best interpreted as model-derived mechanistic frameworks rather than direct evidence of human disease biology.

Therefore, current animal genetic models and 2D epithelial culture systems both exhibit pronounced gaps in fine-tuning cell lineages and establishing co-cultures of multiple microbial, epithelial, and immune components. This limits the depth of mechanistic insights into how M cells intersect with the gut microbiota in chronic inflammation and constrains the reliable evaluation of potential intervention strategies. To compare the complementary utilities, representative readouts, and major limitations of currently available platforms, the principal experimental models used to study intestinal M cells are summarized in Table 1.

**TABLE 1:** Comparative summary of the main experimental models used to study intestinal M cells.

Model type	Representative readouts	Main strengths	Main limitations	Key references
Animal models	M-cell abundance and maturation markers (e.g., GP2, Spi-B, Sox8), Peyer’s patch and follicle-associated epithelium development, antigen uptake and microbial translocation, fecal SIgA, pathogen burden, histologic inflammation, and cytokine responses	Preserve the native *in vivo* mucosal context, including Peyer’s patches, follicle-associated epithelium, immune and stromal compartments, vasculature, microbiota, and systemic inflammatory responses; enable organism-level assessment of antigen sampling, microbial translocation, IgA induction, and inflammation outcomes	Limited ability to distinguish M-cell-specific effects from broader epithelial, lymphoid, or inflammatory alterations; genetic perturbation often disrupts the entire FAE immune-sampling niche rather than selectively targeting M cells; important species differences remain between murine and human M-cell biology; relatively low throughput and limited experimental controllability	Kanaya et al. (2018), Donaldson et al. (2020), Kimura et al. (2020), Nakamura et al. (2020), George et al. (2021)
Conventional 2D systems	Transepithelial transport, particle or bacterial uptake/translocation, TEER, permeability assays, epithelial marker expression, and limited detection of M-cell-like features	Technically simple, accessible, and relatively inexpensive; allow controlled apical exposure, permeability assays, and relatively rapid screening; useful for preliminary assessment of transcytosis-like activity, epithelial transport, and barrier-associated responses	M-cell differentiation efficiency is typically low and unstable, and mature phenotypes are difficult to maintain; lack the three-dimensional architecture and multicellular microenvironment of native follicle-associated epithelium; poorly suited for prolonged or complex host–microbiota co-culture	MASUDA et al. (2011), Lapthorne et al. (2012), Kollmann et al. (2023)
Intestinal organoid models	M-cell marker induction (e.g., GP2, SPIB), apical antigen or bacterial uptake, transcytosis, barrier integrity, epithelial transcriptomic responses, cytokine-associated epithelial programs, and patient-specific responses to microbes or therapeutic agents	Provide a human-relevant and experimentally controllable epithelial platform with multilineage differentiation capacity; support inducible generation of GP2^+^ M cells under defined conditions; enable mechanistic analysis of M-cell differentiation, apical uptake, microbial translocation, barrier responses, and drug, dietary, or metabolite effects; adaptable to patient-derived and personalized studies	Lack key non-epithelial components, including immune cells, vasculature, lymphatics, and enteric neural networks; microbial co-culture remains technically challenging, especially for anaerobic species and long-term community stability; results are sensitive to ECM composition, differentiation window, and exposure format; expression of markers such as GP2 or SPIB alone does not necessarily indicate full functional equivalence to *in vivo* M cells	Sato et al. (2009), Rouch et al. (2016), Wood et al. (2016), Ding et al. (2020), Puschhof et al. (2021), Fofanova et al. (2024), Ballerini et al. (2025), Capeling et al. (2026)

FAE, follicle-associated epithelium; GP2, glycoprotein 2; SIgA, secretory immunoglobulin A; SPIB, Spi-B transcription factor; TEER, transepithelial electrical resistance.

### 1.5 Recent advances in human intestinal organoid–based epithelial–microbe interaction models

A seminal study by Sato et al. demonstrated that a single Lgr5^+^ intestinal stem cell can self-organize *in vitro* into a crypt–villus–like unit (Sato et al., 2009), human intestinal organoids have become a robust epithelial reconstruction platform that can be expanded long term while retaining lineage differentiation capacity. This provides a reproducible experimental basis to dissect epithelial-intrinsic programs without confounding inputs from immune or stromal cells.

In the IBD context, early patient-derived intestinal epithelial organoid studies tested whether inflammation-associated phenotypes persist *ex vivo* and how these phenotypes relate to stem-cell compartment plasticity and “inflammatory memory” (Dotti et al., 2017; Noben et al., 2017). This line of work converted epithelial contributions that are difficult to separate in clinical tissue into experimentally controllable, causal questions. Subsequent systematic profiling of colonic organoids from IBD patients further characterized morphological and barrier defects, and reproduced key inflammatory phenotypes in control organoids using defined pro-inflammatory cytokine combinations, strengthening a controllable link between the inflammatory microenvironment, epithelial phenotypes, and drug responses (d’Aldebert et al., 2020).

Over the past few years, several major technical barriers for epithelial–microbe modeling—including limited access to the luminal surface, difficulty maintaining anaerobic niches, and the lack of physiological shear stress and oxygen gradients—have been addressed through multiple approaches. Polarity-reversed “apical-out” organoids enabled direct luminal exposure for infection and apical stimulation experiments (Co et al., 2021). Hypoxia-tolerant apical-out human small-intestinal organoids under low-oxygen conditions enabled co-culture with anaerobic bacteria, moving toward microbial colonization and barrier assessment under oxygen tensions closer to *in vivo* conditions (Kakni et al., 2023). Modular organoid–microbe co-culture devices further lowered the technical threshold for building human epithelial–microbiota interaction systems (Fofanova et al., 2024).

In parallel, transcriptional response networks mapped in human colonic epithelial organoids under multi-cytokine stimulation supported an epithelial response framework for molecular stratification in IBD, helping shift inflammation research from descriptive phenotypes to quantifiable signaling networks (Pavlidis et al., 2022). Tissue-engineered platforms integrating human fecal samples, peristalsis-like mechanical stimulation, and chip-based microenvironments improved the external validity of microbiota–host interaction models (Ballerini et al., 2025). Methodological advances combining donor mixing with single-cell multi-modal readouts enabled construction of “response dictionaries” for epithelial programs induced by secreted niche factors and computational mapping onto IBD single-cell/spatial atlases, moving organoids toward quantitatively comparable platforms for mucosal microenvironment responses (Capeling et al., 2026). Consistently, patient-derived intestinal epithelial organoid biobanks combined with inflammatory stimulation using bacterial lysates revealed inter-individual differences in epithelial innate immune gene modules and disease heterogeneity, providing a resource base for subsequent drug screening and precision stratification (Shojaei Jeshvaghani et al., 2026).

### 1.6 M cell-enriched intestinal organoids: From differentiation pathways to regulatory mechanisms

In murine systems, Lgr5^+^ intestinal stem cell-derived small intestinal organoids retain multilineage differentiation potential and spontaneously form crypt-villus-like structures in the presence of extracellular matrix and growth factors, thereby reconstructing a variety of epithelial cell types (Yui et al., 2012). This enables researchers to dissect M cell differentiation pathways at the pure epithelial level without disturbing lymphoid tissue architecture.

Further studies have revealed intrinsic negative regulatory mechanisms controlling M cell density and differentiation. In murine studies, M cells themselves express osteoprotegerin (OPG), a soluble RANKL antagonist that inhibits neighboring FAE from differentiating into M cells by blocking the RANKL–RANK interaction. In OPG-deficient mice, M cell numbers are markedly increased, accompanied by enhanced commensal-specific IgA responses. However, these mice display higher susceptibility to invasive pathogens such as *Salmonella*, indicating that OPG-mediated feedback inhibition is physiologically important for maintaining appropriate M cell abundance and immune homeostasis (Kimura et al., 2020).

Beyond OPG, transcriptional regulatory networks also restrict M cell numbers. Using murine organoid and mouse models, George et al. demonstrated using organoid and mouse models that the transcription factor Atoh8 influences M cell density through PRC2-mediated mechanisms. Atoh8-deficient mice displayed a significant increase in GP2^+^ M cells with upregulated expression of key M cell-related genes such as Spi-B and Sox8. Moreover, organoids derived from these mice exhibited higher expression of mature M cell markers following RANKL treatment, suggesting that Atoh8 functions as a negative regulator of M cell differentiation (George et al., 2021).

Furthermore, murine studies suggest that host age-related changes affect M cell number and phenotype. In aged mice, GP2^+^ M cells in Peyer’s patches are significantly reduced. Colonization with microbiota from young mice or stimulation with flagellin can partially restore M cell numbers and function, indicating that the microbiota contributes to age-related decline of M cells (Donaldson et al., 2020).

Patient-derived intestinal organoids have been shown to retain disease-specific profiles and reflect clinical therapy responses in IBD, enabling *in vitro* drug screening that correlates with patient outcomes (Frias et al., 2023). Organoid-derived monolayer systems further facilitate evaluation of drug metabolism and absorption relevant to pharmacotherapy discovery, underscoring the translational potential of these platforms (Inui et al., 2024). Importantly, M cell–enriched patient-derived intestinal organoids may provide a functional platform to evaluate individual responses to microbes or therapeutic agents. This approach may support patient-level treatment stratification beyond conventional clinical markers. In human systems, conditions for inducing M cells have been successfully established in human small intestinal organoids. By using human intestinal organoids combined with retinoic acid and lymphotoxin signals, GP2^+^ human M cells can be induced, providing a basis for extending M cell research to patient-derived materials and clinically relevant studies (Ding et al., 2020).

Taken together, these studies indicate that intestinal organoids are not only controllable systems for generating M cells, but that by integrating differentiation signals with intrinsic negative regulators (such as OPG and Atoh8), M cell density and functional states can be finely tuned. This provides an important experimental foundation for reconstructing microbe–epithelium interactions *in vitro*, dissecting M cell regulatory networks, and evaluating intervention strategies.

### 1.7 Gut Microbiota–M cell–organoid platforms: A controllable experimental space for drugs and strains

#### 1.7.1 Microbe-mediated direct M cell regulation

Emerging evidence from preclinical models suggests that microbial signals may directly modulate M-cell differentiation and function, although direct validation in human systems remains limited. In support of this concept, a murine proof-of-concept study showed that Pasteurized *Akkermansia muciniphila* (pAKK) significantly increases the number of GP2^+^ mature M cells in Peyer’s patches of mice, accompanied by higher *Salmonella* colonization levels in Peyer’s patches, mesenteric lymph nodes, and the spleen. In that study, pAKK induces M cell differentiation through its TLR2 ligands, activating the TLR2–MyD88–NF-κB pathway and bypassing the requirement for classical RANKL signaling, suggesting that the gut microbiota can directly drive M cell differentiation via TLR pathway (Zhang et al., 2025). This study indicates that some candidate probiotics may, in certain contexts, enhance pathogen susceptibility by modulating M cells. M cell-enriched organoid systems thus represent an ideal pre-screening tool for evaluating the safety and mechanisms of action of microbial strains. Representative bacterial species, microbial products, and selected microbial metabolites reported to interact with or modulate intestinal M cells are summarized in Table 2.

**TABLE 2:** Representative bacterial species and microbial products reported to interact with or modulate intestinal M cells.

Factor	M-cell-related mechanism	Functional implication	References
FimH-positive Gram-negative bacteria	GP2 on apical M cells recognizes FimH, promoting bacterial uptake and transcytosis across the follicle-associated epithelium	Supports mucosal antigen sampling but also provides a route for microbial entry	Kimura et al. (2020)
Adherent-invasive *Escherichia coli* (AIEC)	Preferentially exploits Peyer’s patch M cells for translocation into underlying lymphoid tissue in an LPF-dependent manner	Supports a role for M cells as a disease-relevant epithelial gateway in ileal Crohn’s disease	Chassaing et al. (2011)
Pasteurized Akkermansia muciniphila (pAKK)	Promotes GP2-positive M-cell differentiation through TLR2-MyD88-NF-κB signaling	Indicates that microbiota-derived interventions may enhance M-cell maturation in a context-dependent manner	Zhang et al. (2025)
Bacterial flagellin	Partially restores age-associated decline in GP2-positive M cells and improves M-cell functional maturation	Suggests that microbial signals contribute to maintenance of M-cell fitness and mucosal immune surveillance	Donaldson et al. (2020)
*Brucella abortus*	PrP(C)-dependent uptake by intestinal M cells; Hsp60 implicated as a PrP(C)-binding bacterial factor in the broader *Brucella* literature	Expands the receptor-level framework of M-cell-mediated pathogen entry beyond the GP2 pathway	Watarai et al. (2003), Nakato et al. (2009), 2012
*Yersinia* enterocolitica	β1-integrin-associated apical adhesion, internalization, and translocation in M-like cell models; invasin-dependent uptake is classically described, although additional adhesins may contribute	Represents a classical pathogen-entry mechanism showing how invasive bacteria exploit M-cell-like uptake pathways	Schulte et al. (2000), Hamzaoui et al. (2004)

AIEC, adherent-invasive *Escherichia coli*; CD, Crohn’s disease; GP2, glycoprotein 2; LPF, long polar fimbriae; PrP(C), cellular prion protein; Hsp60, heat shock protein 60.

However, moving from *in vivo* observations to *in vitro* causal testing with reproducible, quantitative readouts still requires overcoming key platform-level technical barriers. In organoid systems, the main limitations are that the M-cell lineage is difficult to generate in a sustained and stable manner, and microbial exposure to the luminal (apical) side is hard to make both reliably accessible and tightly parameter-controlled, which constrains reproducibility in mechanistic validation (Min et al., 2020). On the induction side, in murine organoid systems, RANKL can rapidly induce SPIB and initiate the M-cell differentiation program in 3D crypt-like organoids derived from Lgr5^+^ intestinal epithelial stem cells, leading to upregulation of maturation markers such as GP2 (de Lau et al., 2012). In addition, evidence from murine organoid and preclinical studies suggests that canonical NF-κB activators in inflammatory microenvironments, such as TNFα, can enhance the output of RANKL-dependent M-cell gene modules, making organoid M-cell–like states more closely match the induction dynamics observed in FAE *in vivo* (Wood et al., 2016). In human systems, combined retinoic acid and lymphotoxin signaling has been used to establish a more reliable protocol for differentiating GP2^+^ human M cells, enabling subsequent assessment of apical antigen/pathogen uptake and trans-epithelial transport (Ding et al., 2020).

On the exposure side, standard 3D organoids embedded in Matrigel or BME typically adopt a basal-out/apical-in polarity, with the apical luminal surface enclosed within a sealed cyst. This configuration makes it difficult for microbes to interact with the M-cell apical membrane in a physiologically relevant context (apical receptors, glycocalyx, and brush-border microenvironment), thereby limiting quantitative measurement of transcytosis and bacterial translocation. Two main strategies are used to address this structural constraint. First, microinjection can deliver a defined dose of a single strain or a complex community into the organoid lumen, achieving apical exposure while preserving epithelial polarity and barrier architecture. The relatively low-oxygen luminal environment can also support short-term survival and expansion of some anaerobes, making this approach suitable for capturing early microbe–epithelium/M-cell interaction events and transport-related readouts (Wilson et al., 2015; Williamson et al., 2018; Puschhof et al., 2021). Second, polarity reversal (apical-out) can be induced by removing extracellular matrix support and culturing organoids in suspension, exposing the apical surface outward. This greatly reduces the need for single-organoid injection and increases throughput for strain and small-molecule screening; however, it is important to evaluate potential effects of polarity remodeling on lineage composition, tight-junction integrity, and stress responses to avoid misattributing configuration-driven biases to microbe-specific effects (Co et al., 2019). In addition, establishing organoid-derived epithelium as Transwell monolayers provides direct apical access and can be coupled with M-cell–like differentiation protocols (e.g., RANKL plus TNFα), enabling parallel measurements of transport, barrier function, and inflammation-related transcriptomic outputs under standardized exposure conditions, and supporting drug-combination and multi-condition control designs (Fasciano et al., 2019; Puschhof et al., 2021).

Therefore, for murine observations such as pAKK-associated M-cell remodeling that may be driven through a TLR2–MyD88–NF-κB axis, a modular experimental framework—built on robust M-cell enrichment and combined with controlled apical microbial exposure (microinjection, apical-out, or monolayers) and multi-dimensional readouts of transport, barrier integrity, and inflammatory signaling—should better distinguish true M-lineage effects from secondary changes caused by non-specific inflammation or barrier injury.

#### 1.7.2 Dietary antigens and M cell permeability

Diet not only shapes microbiota composition, thereby influencing mucosal immunity, but also provides a large pool of luminal antigens. Under normal conditions, most dietary antigens are degraded in the intestinal lumen, and only a small proportion is sampled by M cells to induce oral tolerance. Patients with IBD often exhibit intolerance to certain dietary antigens and exaggerated immune responses, suggesting that dietary components may excessively stimulate the immune system via M cell pathways. A typical example is the impact of food additives, such as emulsifiers. Common emulsifiers, including carboxymethyl cellulose (CMC) and polysorbate 80 (P80), could reduce gut microbial diversity and impair the mucus layer in mouse models, inducing colitis in susceptible hosts (Chassaing et al., 2015). In contrast, high-fiber diets and certain prebiotics can reduce bacterial adhesion and translocation across the epithelium, possibly by promoting mucus-producing bacteria, increasing barrier thickness, and fostering a more tolerogenic immune environment (Levine et al., 2019). Furthermore, in a human Peyer’s patch model, P80 promotes AIEC translocation across M cells, whereas soluble psyllium fiber inhibits this process (Roberts et al., 2010).

Translating these findings to M cell-containing organoid systems would allow for the systematic testing of how different dietary components or additives (under defined microbial conditions) affect “microbiota-regulated M cell function, epithelial permeability, and inflammatory gene expression,” without relying on whole-animal experiments, which could support the design of dietary strategies that are more “friendly” to the M cell axis.

#### 1.7.3 Functional interfaces with current IBD therapies

Several clinical cohort studies have reported that baseline gut microbiota features significantly influence response rates to biologics such as anti-TNFα agents (Lee et al., 2021) Given that TNFα promotes intestinal M cell differentiation, anti-TNFα therapy is likely to reduce M cell-dependent antigen load by simultaneously suppressing inflammation and inhibiting aberrant M cell generation (Bennett et al., 2016). However, existing studies are largely descriptive, and it remains unclear whether the clinical efficacy of anti-TNFα agent is mediated through direct suppression of inflammation-induced M cell differentiation and antigen influx, or whether the observed reduction in M cells simply reflects secondary effects of global inflammation control. In M cell-enriched patient-derived organoids co-cultured with the microbiota of the patients, the addition of anti-TNFα or other small molecules (such as JAK inhibitors or S1P modulators) offers an opportunity to capture functional interactions among therapy, microbiota, and M cells *in vitro*. This may provide quantitative readouts for personalized treatment decisions, beyond reliance on gross microbiota diversity indices alone.

#### 1.7.4 Microbial metabolites and small molecule screening

Microbial metabolites (particularly SCFAs, e.g., butyrate) reportedly retain the capacity to improve colitis and remodel immune responses in various models, and are considered as “postbiotics” with a therapeutic potential (Ventura et al., 2024). The M-cell-enriched organoid platform could be applied to screen strains or metabolites that could reduce the transmembrane movement of harmful bacteria mediated by M cells, while enhancing the barrier and inducing tolerable IgA. Moreover, thiauto-cos platform could be used to rule out at an early stage those candidates that display anti-inflammatory effects but significantly increase M cell numbers or promote pathogen translocation.

## 2 Discussion

More broadly, the interpretation of M-cell biology in IBD remains constrained by the fact that many mechanistic insights derive from murine or reductionist systems, whereas direct human evidence remains comparatively limited. Despite the clear advantages of M cell-enriched intestinal organoid models in enabling the controlled investigation of epithelial–microbial interactions, several limitations should be acknowledged. A key constraint of the current organoid-based systems is the lack of immune cell populations and vascular components, including lymphatic and blood vessels, being essential for antigen presentation, immune cell trafficking, and signal integration downstream of M cell-mediated transcytosis. In addition, most epithelial organoid systems lack enteric nervous system–related neuronal and glial networks, as well as circuits that can reproduce peristalsis-like mechanical stimulation and neuro–immune regulation. This limits how closely these models can approximate the multi-scale regulatory environment *in vivo* (Childs et al., 2025). At the same time, patient-derived organoids show substantial variability across donors and across sampling sites. Their baseline lineage composition and the magnitude of responses to inflammatory or infectious stimuli can be donor dependent. This variability is a major strength for individualized modeling, but it also becomes a source of bias that must be quantified and controlled by stratification when conclusions are generalized (Jang et al., 2022; Laudadio et al., 2024). A related practical limitation is the challenge of inter-laboratory reproducibility and standardization. Organoid culture outcomes are often affected by ECM batch-to-batch differences, media formulations and differentiation windows, and variation in exposure methods and readout workflows, leading to phenotypic drift within and between batches and reduced comparability (Criss et al., 2021; Kim et al., 2026). In addition, the technical complexity of organoid and co-culture systems can amplify the impact of operational variation on results. This is especially important in multi-component setups, where it is necessary to predefine quality-control metrics and acceptable ranges of process variation (Lee et al., 2024; Kim et al., 2026). It is also important to note that the choice of functional readouts is itself a key constraint on model interpretability. Upregulation of markers such as GP2 or SPIB alone does not necessarily mean that in vivo–level antigen sampling and trans-epithelial transport capacity has been achieved. Maturity and comparability should instead be defined using quantitative uptake and transport flux measurements, epithelial barrier readouts, and functional endpoints that link to downstream immune activation (Lewis et al., 2024; Wang et al., 2025). In addition, current organoid–microbiota co-culture systems face important limitations in maintaining microbial complexity and long-term stability, as many anaerobic or fastidious species are difficult to sustain under *in vitro* conditions. As a result, co-cultured microbial communities are often simplified or biased, which may limit the ability of these models to fully recapitulate the dynamic and competitive ecosystem of the human gut. As a result, certain aspects of mucosal immune activation (e.g., antigen-specific T- and B-cell responses, IgA class switching, and lymphatic drainage) could not yet be fully recapitulated in these reductionist epithelial models.

Nevertheless, rapid advances in co-culture technologies provide promising avenues to overcome these limitations. Emerging strategies are beginning to integrate intestinal organoids with immune cells and with endothelial networks to support vascularization, laying the groundwork for multi-component co-culture models (Recaldin et al., 2024; Wen et al., 2024). Advances in microfluidic organoid-on-a-chip systems further promise enhanced physiological relevance by enabling perfusion and dynamic environmental control. Microfluidic gut chips can recreate key features of the intestinal lumen through continuous flow, shear stress, and mechanical stretch, allowing week-long epithelial–microbe co-culture while maintaining an anaerobic niche (Kim et al., 2012; Sasan et al., 2019). In addition, scaffold-guided approaches that generate perfusable, tubular “mini-intestines” with an open, accessible lumen further extend conventional closed-cyst organoids into host–microbe interaction platforms that support long-term microbial colonization (Nikolaev et al., 2020). Incorporating these approaches into M cell–focused models might allow for a more comprehensive reconstruction of the gut microbiota–M cell–immune axis, thereby strengthening the translational value of organoid-based platforms for studying IBD pathogenesis and for screening microbiota-targeted or mucosa-directed therapeutic. Notably, the temporal resolution of current *in vitro* platforms should be considered when extrapolating organoid-based findings to chronic intestinal inflammation. Static organoid–microbiota co-culture systems are typically limited to short-term exposure (usually within 24 h), whereas microfluidic gut-on-a-chip models permit extended and dynamically regulated host–microbe interactions (Poletti et al., 2020). This constraint should be taken into account when interpreting M-cell–microbiota responses observed *in vitro*, particularly in the context of chronic inflammatory conditions such as IBD.

However, M cell-containing organoid models can be used for high-throughput screening of beneficial strains or metabolites: by examining the effects of different microbes or their supernatants on M cells and epithelial immune gene expression in organoids, researchers can rapidly identify candidate probiotics or postbiotic factors with anti-inflammatory and immunomodulatory potential. In contrast, organoid models could allow for preliminary assessment of how new drug candidates affect the mucosal barrier and immune responses. Looking ahead, combining patient-derived intestinal organoids with autologous gut microbiota to establish “patient-specific” models may enable individualized drug screening.

In summary, the gut microbiota, through M cell modulation, represents a double-edged sword in mucosal immunity. On the one hand, it could promote immune surveillance and pathogen defense. On the other hand, when imbalanced, it might become a trigger for inflammation. In-depth investigation of the microbiota–M cell interactions would not only enhance our understanding of the pathogenesis of IBD and other intestinal inflammatory diseases, but also pave the way for microbiota-based therapeutic strategies. The integrated application of organoids, bioengineering, and multi-omics technologies would potentially offer more comprehensive and precise perspectives for exploring the intestinal “microecology–immunity” network, providing a scientific basis for developing more effective and safer IBD therapies.
